# A Preclinical Model for the ATLL Lymphoma Subtype With Insights Into the Role of Microenvironment in HTLV-1-Mediated Lymphomagenesis

**DOI:** 10.3389/fmicb.2018.01215

**Published:** 2018-06-13

**Authors:** Mattia Vicario, Adriana Mattiolo, Barbara Montini, Maria Assunta Piano, Ilaria Cavallari, Alberto Amadori, Luigi Chieco-Bianchi, Maria Luisa Calabrò

**Affiliations:** ^1^Immunology and Molecular Oncology, Veneto Institute of Oncology, IOV – IRCCS, Padua, Italy; ^2^Department of Surgery, Oncology and Gastroenterology, University of Padova, Padua, Italy

**Keywords:** HTLV-1, ATLL, lymphoma, microenvironment, fibroblasts, preclinical model

## Abstract

Adult T cell Leukemia/Lymphoma (ATLL) is a mature T cell malignancy associated with Human T cell Leukemia Virus type 1 (HTLV-1) infection. Among its four main clinical subtypes, the prognosis of acute and lymphoma variants remains poor. The long latency (3–6 decades) and low incidence (3–5%) of ATLL imply the involvement of viral and host factors in full-blown malignancy. Despite multiple preclinical and clinical studies, the contribution of the stromal microenvironment in ATLL development is not yet completely unraveled. The aims of this study were to investigate the role of the host microenvironment, and specifically fibroblasts, in ATLL pathogenesis and to propose a murine model for the lymphoma subtype. Here we present evidence that the oncogenic capacity of HTLV-1-immortalized C91/PL cells is enhanced when they are xenotransplanted together with human foreskin fibroblasts (HFF) in immunocompromised BALB/c Rag2^-/-^γ_c_^-/-^ mice. Moreover, cell lines derived from a developed lymphoma and their subsequent *in vivo* passages acquired the stable property to induce aggressive T cell lymphomas. In particular, one of these cell lines, C91/III cells, consistently induced aggressive lymphomas also in NOD/SCID/IL2Rγ_c_ KO (NSG) mice. To dissect the mechanisms linked to this enhanced tumorigenic ability, we quantified 45 soluble factors released by these cell lines and found that 21 of them, mainly pro-inflammatory cytokines and chemokines, were significantly increased in C91/III cells compared to the parental C91/PL cells. Moreover, many of the increased factors were also released by human fibroblasts and belonged to the known secretory pattern of ATLL cells. C91/PL cells co-cultured with HFF showed features reminiscent of those observed in C91/III cells, including a similar secretory pattern and a more aggressive behavior *in vivo*. On the whole, our data provide evidence that fibroblasts, one of the major stromal components, might enhance tumorigenesis of HTLV-1-infected and immortalized T cells, thus throwing light on the role of microenvironment contribution in ATLL pathogenesis. We also propose that the lymphoma induced in NSG mice by injection with C91/III cells represents a new murine preclinical ATLL model that could be adopted to test novel therapeutic interventions for the aggressive lymphoma subtype.

## Introduction

According to the criteria of the World Health Organization [WHO] (2008) for classification of tumors originating from hematopoietic and lymphoid tissues, Adult T cell Leukemia/Lymphoma (ATLL) is defined as a peripheral T cell neoplasia linked to infection with Human T cell Leukemia Virus type 1 (HTLV-1), a virus identified first by Gallo and co-workers in 1980 (Poiesz et al., 1980). ATLL incidence is therefore linked to the prevalence of HTLV-1 infection, which is not uniformly distributed around the world; clusters of high HTLV-1 prevalence are mainly found in Southwestern Japan, the Caribbean Basin and some parts of Africa and South America (Gessain and Cassar, 2012). ATLL develops almost exclusively in adults, although a few cases in children have been reported (de Oliveira et al., 1990; Watanabe, 2017). The diagnosis is mainly based on clinical features, presence of HTLV-1 infection and, when detected, peculiar tumor cell morphology, represented by atypical circulating lymphocytes with petal-shaped nuclei (Matutes, 2007). From the clinical point of view, ATLL is characterized by a high heterogeneity in its presentation and clinical course. Four main subtypes have been described (Shimoyama, 1991), two aggressive and fast-growing (acute leukemia, lymphoma) and two indolent (smoldering, chronic) variants. A new classification has recently been proposed that combines clinical features with specific genetic aberrations shown to have a prognostic value (Kataoka et al., 2018). Treatment strategies are based on ATLL subtype, and antiviral therapy is usually effective in slowly progressing subtypes. Prognosis of fast-growing variants remains poor in spite of various attempts of pharmacologic treatments (Hermine, 2015; Katsuya et al., 2015).

The very prolonged latent period (decades) between primary infection and development of full-blown disease and the relative rare occurrence of ATLL among asymptomatic HTLV-1 carriers (3–5%) suggest that additional genetic and/or epigenetic changes in infected T cells as well as host factors are necessary for ATLL induction. Besides ATLL, HTLV-1-infected subjects may also feature opportunistic infections (Nakada et al., 1987) and develop chronic inflammatory disease such as myelopathy (Gessain et al., 1985; Osame and Igata, 1989), arthropathy, myositis, uveitis, and dermatitis (Yamano and Sato, 2012). Thus, circumstantial evidence indicates that the infection with HTLV-1 induces deficiency and dysregulation of host immunity.

HTLV-1 belongs to the deltaretrovirus genus, together with the simian T lymphotropic virus and bovine leukemia virus. HTLV-1 is a complex retrovirus whose genome comprises structural, regulatory and accessory genes. Accumulating evidence indicates that the regulatory gene *tax* is crucially involved in ATLL pathogenesis. In fact, Tax protein exhibits pleiotropic functions (Romanelli et al., 2013); besides transcriptionally activating its long terminal repeats (Felber et al., 1985; Seiki et al., 1986), it interacts with cellular transcription factors (NF-kB, CREB, and AP-1) and upregulates the expression of multiple cellular genes involved in cell proliferation and genomic instability (Armstrong et al., 1993; Baranger et al., 1995; Munoz and Israel, 1995; Fujii et al., 2000; Grassmann et al., 2005; Fochi et al., 2018). However, in the majority of cases, ATLL cells show a Tax-low or Tax-negative phenotype, suggesting that Tax, while critical for T cell immortalization and transformation, may be not crucial in late stages of ATLL (Takeda et al., 2004). In contrast, another viral gene, the HTLV-1 basic leucine zipper factor (HBZ) encoded in the minus strand of the viral genome, appears to be transcribed in all cases of ATLL (Gaudray et al., 2002). Furthermore, it has been reported that HBZ mRNA, but not HBZ protein, could induce T cell proliferation and promote cell survival (Satou et al., 2006). Thus, a current hypothesis is that transactivation by Tax is needed to initiate the processes that lead to ATLL, while HBZ is responsible for maintaining the neoplastic phenotype of ATLL cells (Matsuoka and Jeang, 2007; Giam and Semmes, 2016).

Bidirectional communication of potentially oncogenic cells with surrounding stroma creates a tissue microenvironment permissive to disease initiation and progression. Among the stromal components, fibroblasts play a prominent role; the tumor-promoting activity of cancer-associated fibroblasts has been extensively studied in epithelial cancers (Olumi et al., 1999; Erez et al., 2010). Within the tumor stroma, fibroblasts, likely through a transforming growth factor beta signaling, acquire an activated phenotype which is mainly characterized by their expression of alpha-smooth muscle actin similar to that observed in the wound healing process (Vaughan et al., 2000). These cells are provided with an armamentarium of released factors that can alter tissue homeostasis, promote angiogenesis, cancer cell proliferation and invasiveness, and recruit immunosuppressive and tumor-promoting cells (Kalluri and Zeisberg, 2006; Mueller et al., 2007; Erez et al., 2010; Bissell and Hines, 2011). Specific bone marrow stromal niches have been identified for leukemia development (Jin et al., 2008; Zhang et al., 2012). Concerning ATLL, *in vitro* interaction with epithelial and fibroblastic cell lines was shown to induce apoptosis resistance in primary ATLL cells and ATLL cell lines (Miyatake et al., 2013, 2015) as well as viral latency (Kinpara et al., 2009), highlighting the role of stromal components in ATLL pathogenesis.

Different preclinical models have been developed to better understand the pathogenesis of ATLL and HTLV-linked degenerative/inflammatory diseases (Panfil et al., 2013). Good models for HTLV-1 infection have been established in non-human primates, rabbits and rats (Dodon et al., 2012; Hajj et al., 2012). However, mice are preferred as they are more manageable and cost-effective models to study the virus/host factors critical for ATLL induction and for evaluation of its specific treatments. Xenografts of ATLL cells or some ATLL-derived cell lines in immunocompromised mice successfully replicated features of ATLL (Parrula et al., 2009). On the other hand, T cells immortalized *in vitro* by HTLV-1 showed no or poor growth, depending mainly on the host constitutive immunodeficiency degree, the innate immunity by natural killer (NK) cell antitumor activity being particularly critical for restraining the engraftment (Ishihara et al., 1992; Feuer et al., 1993, 1995; Imada et al., 1995; Liu et al., 2002).

The aim of the present study was to analyze the evolution of HTLV-1-infected T lymphocytes from the immortalized status, commonly observed in asymptomatic carriers, to the quite rare neoplastic transformation leading to clinically overt ATLL. Among the multiple factors involved in this oncogenic switch, including genetic and epigenetic cell alterations, our attention was focused on the influence of one of the major microenvironment components, specifically fibroblasts. Moreover, we established of a highly lymphomagenic C91/PL-derived cell line that, when xenotransplanted into immunodeficient NSG mice, may constitute a new preclinical mouse model for the lymphoma variant of ATLL.

## Materials and Methods

### Cell Lines

The HTLV-1-immortalized C91/PL cell line was established by co-cultivation of umbilical cord blood T cells with leukemic T cells from an ATLL patient (PL) (Popovic et al., 1983), and was originally obtained from Prof. Robin Weiss (Chester Beatty Laboratories, London). This cell line and its *ex vivo*-derived cell lines (designated as C91/I, C91/II and C91/III) were grown in RPMI 1640 (Sigma-Aldrich, Munich, Germany) supplemented with 10% fetal calf serum (FCS, Gibco, Foster City, CA, United States), 2 mM L-glutamine (Gibco) and 50 μg/mL gentamycin (Sigma-Aldrich) (complete medium). Human foreskin fibroblasts (HFF) were a kind gift from Dr. Abatangelo and Dr. Zavan (University of Padova). HFF were propagated in Dulbecco’s modified Eagle Culture Medium (DMEM, Sigma-Aldrich) supplemented with 10% FCS, 2 mM L-glutamine and 50 μg/mL gentamycin.

Analysis of the clonal T cell receptor (TCR) beta chain and gamma chain gene rearrangements in C91/PL cells and in C91/PL-derived cells was carried out using the IdentiClone^TM^ TCRB + TCRG T cell Clonality Assay (Invivoscribe Technologies, San Diego, CA, United States), according to the manufacturer’s instructions. Short tandem repeat (STR) profile of C91/PL and C91/PL-derived cell lines was carried out with the PowerPlex 18D System (Promega, Madison, WI, United States) using an Applied Biosystems 3130XL genetic analyzer and Genemapper ID Ver. 3.2.1 software (BMR Genomics S.r.l., Padua, Italy). As an STR profile of C91/PL cells is not available in DMSZ, ATCC, and COG databases, C91/PL cells and their more tumorigenic counterparts (C91/II and C91/III) were authenticated by comparing the STR profile obtained with 18 genetic markers to that determined using the C91/PL cells received by the National Institute for Biological Standards and Controls (NIBSC), United Kingdom. The presence of the HTLV-1 provirus was confirmed in all *ex vivo* cell lines by qualitative single-round PCR with primer pairs specific for the *tax* region as previously reported (Calabro et al., 1993). All cell lines were mycoplasma-free, as confirmed by periodical PCR check.

### Mice

BALB/c Rag2/Common γ chain (γ_c_)–double KO (Rag2^-/-^γ_c_^-/-^) mice were originally received from the Freiburg University Medical Center, Germany. Null mutation of the RAG2 gene prevents B and T lymphocyte development in these mice, while absence of the cytokine receptor common gamma chain, required for signal transduction of multiple cytokines [including interleukin (IL)-2, IL-4, IL-7, IL-9, IL-15, and IL-21] prevents NK cell maturation. Mice were inbreed and maintained in our animal facility, and were used in the first set of experiments and for the *in vivo* passages of the C91/PL-derived cells. Because of a temporary closure of our animal SPF facility for enlargement and renovation, all mouse colonies were eliminated and subsequently, after a 7 month-gap, restored with new stocks. Therefore, the second set of experiments was conducted with BALB/c Rag2^-/-^γ_c_^-/-^ (BRG) mice (“excluded flora,” i.e., free of rodent pathogens and seven selected opportunistic pathogens as well as Segmented Filamentous Bacteria) obtained from Taconic (Germantown, NY). Other experiments were performed using NOD/SCID/IL2Rγ_c_ KO (NSG) mice, obtained from Charles River (Charles River Laboratories, Calco, Italy), that combine the NOD/SCID background to the lack of a functional common gamma chain.

### Ethics Statement

This study was carried out in accordance with the institutional guidelines that comply with the Italian Animal Welfare Law (D.L. No. 116/1992; and subsequent documents). The project was evaluated and approved by the local ethics committee of the University of Padova (Comitato Etico di Ateneo per la Sperimentazione Animale, CEASA) and communicated to the relevant Italian authority (Italian Ministry of Health, VI Office) (Project No. 32/2009; Permit Protocol No. 51740, 15/09/2010). The project had to be renewed, and it was evaluated and approved by the Italian Ministry of Health (Project and Permit protocol No. 932/2016, 10/10/2016).

### Assessment of the Contribution of Fibroblasts to Lymphoma Growth *in Vivo*

Exponentially growing HFF and C91/PL cells mixed in a 1:2 ratio were suspended in a final density of 6 × 10^7^ total cells/mL, and 0.1 mL of this cell suspension (i.e., 2 × 10^6^ HFF and 4 × 10^6^ C91/PL) was injected intraperitoneally (i.p.) into each mouse. Control mice were i.p. injected with 4 × 10^6^ C91/PL cells. Mice were checked biweekly for cachexia and presence of abdominal masses. For ethical reasons, tumor-bearing animals were killed when presenting signs of suffering, and each mouse was considered to have died from tumor progression on this date. At necroscopy, the finding of organ involvement, abdominal and pelvic tumor masses were considered lymphoma growth, which was subsequently confirmed by histological examination.

### Assessment of Engraftment and Tumorigenesis of C91/PL-Derived Cell Lines

Fragments of masses from Rag2^-/-^γ_c_^-/-^ mice affected by lymphomatous growth were processed under sterile conditions and the obtained cell suspension was used to set up *ex vivo* cultures. After *in vitro* growth for 3–4 weeks, these cells were used as inoculum to assess their ability to induce a lymphomatous growth in mice. Different cell lines were thus obtained from the subsequent *in vivo* passages: C91/I, C91/II and C91/III, corresponding to the first, second and third *in vivo* passage, respectively. Each cell line was then tested for lymphoma induction in different doses and ages of injected mice.

Engraftment efficiency and tumorigenesis of C91/III cells were also assessed in NSG mice. Six-day-old mice were i.p. injected with 4 × 10^6^ C91/III cells/mouse. Four-week-old mice were injected with 1 × 10^6^ and 4 × 10^6^ C91/III cells/mice (five mice per group). Moreover, we further analyzed the contribution of HFF to lymphoma growth by injecting C91/PL cells after *in vitro* co-culture with HFF. To this end, 5-day-old NSG mice were injected i.p. with 4 × 10^6^ co-cultured C91/PL cells (nine mice). Control mice were injected i.p. with 4 × 10^6^ C91/PL cells (seven mice). Mice were checked, culled and analyzed as reported above.

### Histology and Immunohistochemistry

Fragments of infiltrated murine organs and tissues were fixed in 10% formalin and embedded in paraffin. Sections were cut (4-mm thick) from *ex vivo* samples and prepared for appropriate staining. Hematoxylin–eosin staining was used for histological diagnosis. Sections were immunostained for CD25 (Leica Biosystems, Nussloch, Germany) and for Ki67 (Dako, Glostrup, Denmark), as previously reported (Di Stefano et al., 2008).

### Immunophenotypic and Viral Characterization of C91/PL, C91/III and HFF-Co-cultured C91/PL Cell Lines

C91/PL, C91/III and HFF-co-cultured C91/PL were analyzed for the expression of surface antigens by flow cytometry. The following anti-human monoclonal antibodies were used: fluorescein isothiocyanate (FITC)-conjugated anti-CD1a (BD Pharmingen, Franklin Lakes, NJ, United States), phycoerythrin (PE)-conjugated anti-CD2 (Life Technologies, Carlsbad, CA, United States), Alexa488-conjugated anti-CD3, anti-CD4, and anti-CD25 (Bio-Rad Laboratories, Inc., Hercules, CA, United States), phycoerythrin-Cy5 (PC5)-conjugated anti-CD5 and anti CD-7 (Coulter, Fullerton, CA, United States), FITC-conjugated anti-CD34 (BD Pharmingen), PE-conjugated anti-CD117 (Miltenyi Biotec, Bergisch Gladbach, Germany), Allophycocyanin (APC)-conjugated anti-CD133 (Miltenyi Biotec), A488-conjugated anti-FOXP3 (AbD Serotec, Oxford, United Kingdom), PE-conjugated anti-CD54 (eBioscience, Inc., San Diego, CA, United States), and FITC-conjugated anti-cell adhesion molecule 1 (CADM1, Medical and Biological Laboratories, Nagoya, Japan). Samples were analyzed on a BD LSR II flow cytometer (BD Biosciences, Milano, Italy). All cytofluorimetric data were analyzed using Kaluza Analysis software Ver. 1.3 (Beckman Coulter, Brea, CA, United States).

Quantitative analysis of HTLV-1 transcripts in C91/PL, C91/III and HFF-co-cultured C91/PL cells was performed as previously described (Rende et al., 2011; Cavallari et al., 2013).

### Quantitative Analyses of Soluble Factors

To measure soluble factors released by C91/PL, C91/III and HFF-co-cultured C91/PL cells, cells were seeded at a concentration of 1 × 10^6^/mL. Supernatants were collected after 72 h and centrifuged for 5 min at 200 g. Cell pellets were discarded, and supernatants were centrifuged for 30 min at 2,800 *g* to eliminate cell debris. Supernatants were also collected from the three cell lines kept in culture to evaluate concentration fluctuations and consistency of detectable factors during standard passage. The secretory pattern of HFF was measured by seeding 3 × 10^5^ cells in a six-well plate; supernatants were collected after 72 h and processed as described above. To compare the profile and amount of factors released by all cell lines, a Luminex xMAP approach was used (ProcartaPlex Human Cytokine/Chemokine/Growth Factor Panel 1 96 tests, Affymetrix eBioscience Ltd., Hatfield, United Kingdom) for the multianalyte detection of 45 secreted proteins. This assay detects the following proteins: IL-1 receptor antagonist (IL-1RA), IL-1alpha (IL-1α), IL-1beta (IL-1β), IL-2, IL-4, IL-5, IL-6, IL-7, IL-8/ C-X-C motif chemokine ligand 8 (CXCL8), IL-9, IL-10, IL-12p70, IL-13, IL-15, IL-17A, IL-18, IL-21, IL-22, IL-23, IL-27, IL-31, leukemia inhibitory factor (LIF), tumor necrosis factor alpha (TNFα), TNF beta/lymphotoxin-alpha (TNFβ/LTA), interferon alpha (IFNα), IFN gamma (IFNγ), growth-regulated oncogene-alpha GROα/CXCL1, eotaxin/C-C motif chemokine ligand 11 (Eotaxin/CCL11), IFNγ-induced protein 10 (IP10)/CXCL10, monocyte chemotactic protein-1 (MCP-1)/CCL2, macrophage inflammatory protein-1 alpha (MIP-1α)/CCL3, MIP-1 beta, MIP-1β/CCL4, regulated on activation normal T cell expressed and secreted (RANTES)/CCL5, stromal cell-derived factor 1 alpha (SDF-1α/CXCL12), brain-derived neurotrophic factor (BDNF), granulocyte-macrophage colony-stimulating factor (GM-CSF), hepatocyte growth factor (HGF), placental growth factor (PLGF), epidermal growth factor (EGF), fibroblast growth factor 2 (FGF-2), platelet-derived growth factor-BB (PDGF-BB), stem cell factor (SCF), nerve growth factor beta (βNGF), vascular endothelial growth factor A (VEGF-A), VEGF-D.

IL-8/CXCL8 and TNFα were also quantified using cytokine-specific assays (Human IL-8-ELISA and Human TNFα-ELISA Ready-SET-Go Kits, Affymetrix eBioscience Ltd.), according to the manufacturer’s instructions.

### Analysis of the Crosstalk Between C91/PL Cells and HFF

To assess whether HFF were able to modulate C91/PL cell turnover, 2 × 10^5^ C91/PL cells were co-cultured in the presence or absence of semiconfluent HFF seeded onto a six-well plate. Induced apoptosis was measured after 24, 48, and 72 h of culture by flow cytometry after Annexin V/Propidium Iodide (PI) staining (Annexin-V-FLUOS Staining Kit, Roche Diagnostics, Mannheim, Germany). Apoptosis was induced by serum reduction (5% FCS). Proliferation was analyzed by flow cytometry after staining with carboxyfluorescein succinimidyl ester (Cell Trace^TM^ CFSE, Life Technologies).

Long-term HFF-co-culture of C91/PL cells was carried out in T75 flasks to periodically assess morphological and phenotypic changes. Two-month co-culture of C91/PL cells was carried out, and then cells were removed from co-culture and maintained as long as 6 weeks in culture to ensure the absence of residual fibroblasts. The secretory pattern, the profile of HTLV-1 transcripts and the ability to induce *in vivo* lymphoma of these long-term co-cultured cells were analyzed at this time point.

To assess the contribution of soluble factors in the crosstalk between C91/PL cells and HFF, short-term co-cultures in six-well plates were set up, with C91/PL cells added directly to the HFF or placed into transwell chambers (BD Falcon Cell Culture Inserts, Durham, NC, United States; pore size 0.4 μm). The amount of secreted IL-8/CXCL8 and TNFα was measured in the supernatants collected after 3 and 10 days of co-culture.

### Statistical Analyses

Curves reporting the percentage of survivors over time were estimated by the Kaplan–Meier method and compared with the log-rank test. Data analysis was performed using the MedCalc statistical software (Mariakerke, Belgium). Statistical data are presented as mean ± standard deviation. Two-sided Student’s *t*-test was used to estimate statistical significance of differences between the two cell lines. *P*-values < 0.05 were considered significant.

## Results

### Co-inoculation of Fibroblasts Promotes the Tumorigenesis of C91/PL Cells

Preliminary experiments conducted to xenograft, either i.p. or subcutaneously, the HTLV-1-immortalized C91/PL cell line at different doses (from 2 to 10 × 10^6^ cells per mouse) in immunodeficient adult Rag2^-/-^γ_c_^-/-^ mice did not lead to tumor development (data not shown). The evidence that fibroblasts are effective in promoting tumor initiation and progression in different mouse models of carcinogenesis (Bissell and Hines, 2011) prompted us to analyze whether co-injection of fibroblasts with C91/PL cells in newborn Rag2^-/-^γ_c_^-/-^ mice could facilitate the engraftments of this cell line. Thus, we performed a first set of experiments using six 4–6-day-old Rag2^-/-^γ_c_^-/-^ mice per group: control mice were i.p. injected with 4 × 10^6^ C91/PL cells, whereas the experimental group received the same dose of C91/PL cells mixed with HFF in a 2:1 ratio. This experiment was repeated twice and each experiment was stopped after 200 days. As shown in **Table 1**, all control mice (12 of 12) were disease-free throughout the experiment and autopsy did not disclose any pathological sign. Conversely, among the 12 animals receiving C91/PL cells mixed with HFF, four developed lymphoma with a median survival time of 105 days.

**Table 1:** Lymphomagenic efficiency of C91/PL cells in Rag2^-/-^γ_c_^-/-^ mice.

	No. of positive mice/total No. of mice^a^
C91/PL	0/12
C91/PL + HFF	4/12^b^


**^*a*^4-6-day-old mice were intraperitoneally (i.p.) inoculated with 4 × 10^*6*^ C91/PL alone or with 2 × 10^*6*^ human foreskin fibroblasts (HFF) cells. ^*b*^Mice with lymphomatous abdominal infiltration were culled at 70, 90, 120, and 195 days after i.p. cell inoculation.**

At necroscopy, tumor masses were found in the abdomen, often extending into the pelvic cavity. The tumor infiltrated the mesentery, the small and large intestine, the liver hilum and the pancreas. The spleen was only slightly enlarged and free from lymphoma invasion. Histologically, the neoplastic tissue was composed of large pleomorphic cells with abundant eosinophilic cytoplasm and vesicular basophilic, irregularly lobulated nucleus with prominent nucleoli. Numerous multinucleated syncytial giant cells were also observed and mitotic figures were common. Necrotic and hemorrhagic areas were sometimes found within the lymphomatous masses and organ infiltrates.

Thus, in this set of experiments, using mice deriving from an in-house long-lasting Rag2^-/-^γ_c_^-/-^ colony, co-inoculation of fibroblasts was found to trigger the oncogenic potential of the HTLV-1-immortalized T cell line.

### *Ex Vivo* C91/PL-Derived Cell Lines Induce Aggressive Lymphoma

**FIGURE 1:**
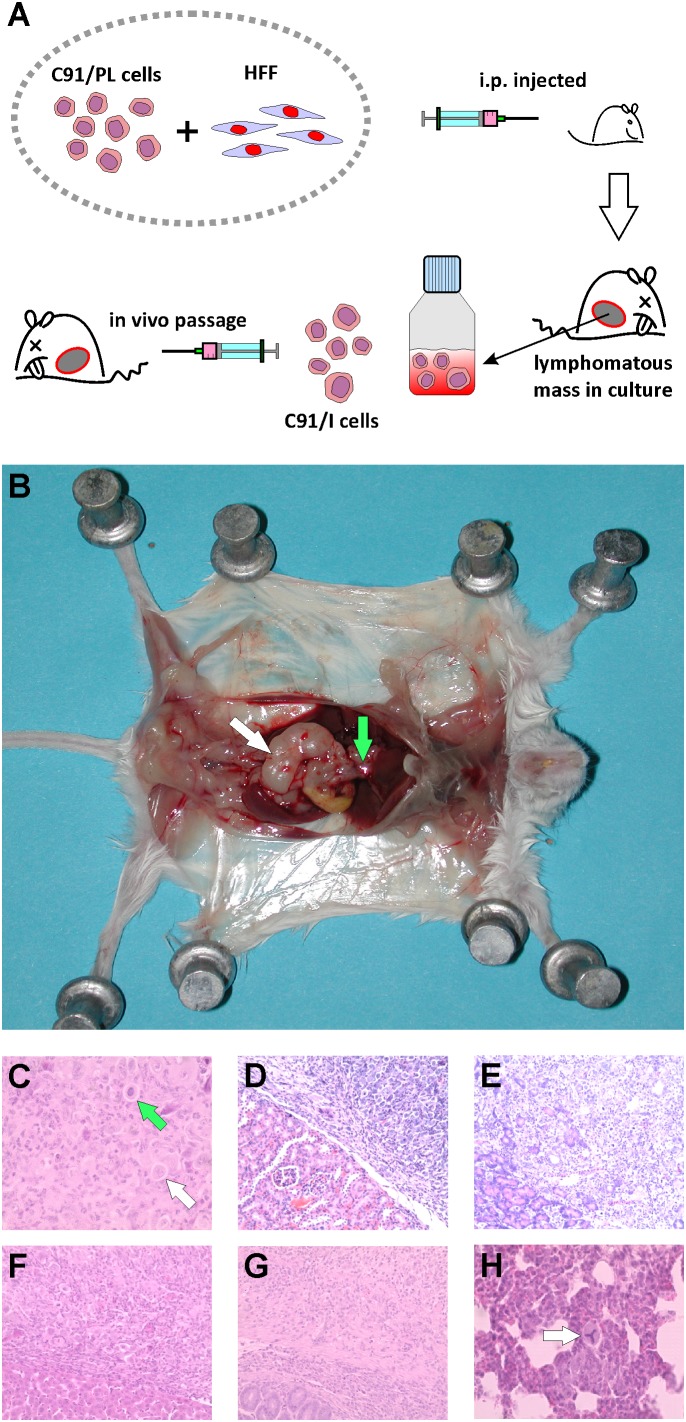
Xenotransplantation of C91/PL-derived cell lines in Rag2^-/-^γ_c_^-/-^ mice. **(A)** Schematic representation of the *in vivo* experiments. C91/PL cells intraperitoneally co-injected with human fibroblasts into a 5-day-old Rag2^-/-^γ_c_^-/-^ mouse lead to lymphoma development. A fragment of the induced abdominal lymphomatous mass was processed under sterile conditions and cultured *in vitro* to establish a C91/PL-derived lymphomatous cell culture (C91/I). Injection of these cells into a Rag2^-/-^γ_c_^-/-^ mouse led to an aggressive Adult T cell Leukemia/Lymphoma (ATLL)-like lymphoma. Additional *in vitro*/*in vivo* passages were done with the establishment of two other lymphomatous cell lines (C91/II and C91/III). **(B)** Macroscopic view of lymphomatous masses developed in a mouse injected with C91/III cells involving mesenteric nodes (white arrow) and the liver hilum (green arrow). **(C)** Hematoxylin and eosin staining of formalin-fixed, paraffin-embedded tissues shows tumor mass composed of pleomorphic cells mixed with syncytial cells (white arrow) with giant nuclei; the green arrow shows a pleomorphic cell with a giant nucleus. Original magnifications 400×. **(D–G)** Lymphomatous infiltration of kidney pericapsular area, pancreas, liver, and intestinal wall, respectively. Original magnification 200×. **(H)** Lung parenchyma with small embolic metastasis; arrow indicates abnormal tripolar mitosis. Original magnification 400×.

The tumor mass from one of the four diseased mice of the first set of experiments, culled 90 days after cell injection, was removed and processed under sterile conditions and a continuous cell culture was obtained (C91/I), as shown in **Figure 1A**. These cells were then i.p inoculated into 4–5-day-old Rag2^-/-^γ_c_^-/-^ mice at different doses and all (7 of 7) animals belonging to the group injected with the higher dose developed a very aggressive and invasive lymphoma with a short latency period (median survival, 15 days, **Table 2**). Two subsequent *in vivo* passages, followed by establishment of tumorigenic cell lines (C91/II and C91/III), were performed. C91/II cells engrafted into mice with high efficiency and also induced aggressive and diffused tumor masses in 2–4-week-old mice (**Table 2**). All mice (10 out of 10) inoculated with 4 × 10^6^ C91/III cells at 4–8 days of age developed a lymphoma with a median latency of 32 days. C91/II and C91/III cell lines showed an increased lung tropism, resulting in frequent lung metastases as well as in lymphomatous lung involvement as primary presentation. Mice inoculated with these cell lines showed a pathological picture similar to that described above and shown in **Figure 1B**. Moreover, involvement by lymphomatous whitish tissue was also observed in the renal pericapsular fatty tissue, with invasion of cortical kidney parenchyma. In some animals, tumor infiltration was also found in ovary, uterine wall and testis. The abdominal and diaphragmatic peritoneal surface was frequently scattered with small neoplastic white nodules and, in two mice, injected with 4 × 10^6^ C91/III cells at 4 days of age, slightly hemorrhagic ascitic fluid was also found in the peritoneal cavity. Furthermore, the muscles surrounding the lumbar spinal column often appeared infiltrated by whitish tumor tissue. Microscopically, mice showed histological and cytological features indicative of lymphomatous growth (**Figure 1C**); kidney, pancreas, liver and intestinal wall were constantly infiltrated (**Figures 1D–G**). Neoplastic white nodules or small metastatic cell embolic aggregates and single neoplastic cells (**Figure 1H**) within capillaries were found in the lung of 10 mice. Immunochemical staining of lung metastases to detect human CD25 (i.e., IL-2 receptor alpha chain) showed the presence of a consistent number of neoplastic cells (**Supplementary Figure S1**). The spleen was free from lymphomatous involvement, being characterized by erythroid and myeloid hyperplasia of the red pulp.

**Table 2:** Lymphoma induction by C91/PL-derived cells in Rag2^-/-^γ_c_^-/-^ mice.

C91/PL-derived cells	Age of mice (days)	No. of cells injected	No. of positive mice/total No. of mice	Median latency (Range, days)
C91/I	4–5	1 × 10^6^	0/4	–
	4–5	4 × 10^6^	7/7	15 (12–34)
C91/II	4–8	4 × 10^6^	15/15	17 (9–41)
	16	4 × 10^5^	3/3	112 (61–112)
	30	4 × 10^5^	3/7	133 (53–202)
	30	4 × 10^6^	3/6	34 (26–34)
C91/III	5	1 × 10^6^	2/3	52 (41–62)
	4–8	4 × 10^6^	10/10	32 (17–62)


These findings indicated that the highly tumorigenic capacity acquired by C91/PL cells following co-transplantation with HFF, and through *in vitro/in vivo* passages, is a stable and reproducible property.

### *In Vivo* Validation of the Supporting Role of Fibroblasts

**FIGURE 2:**
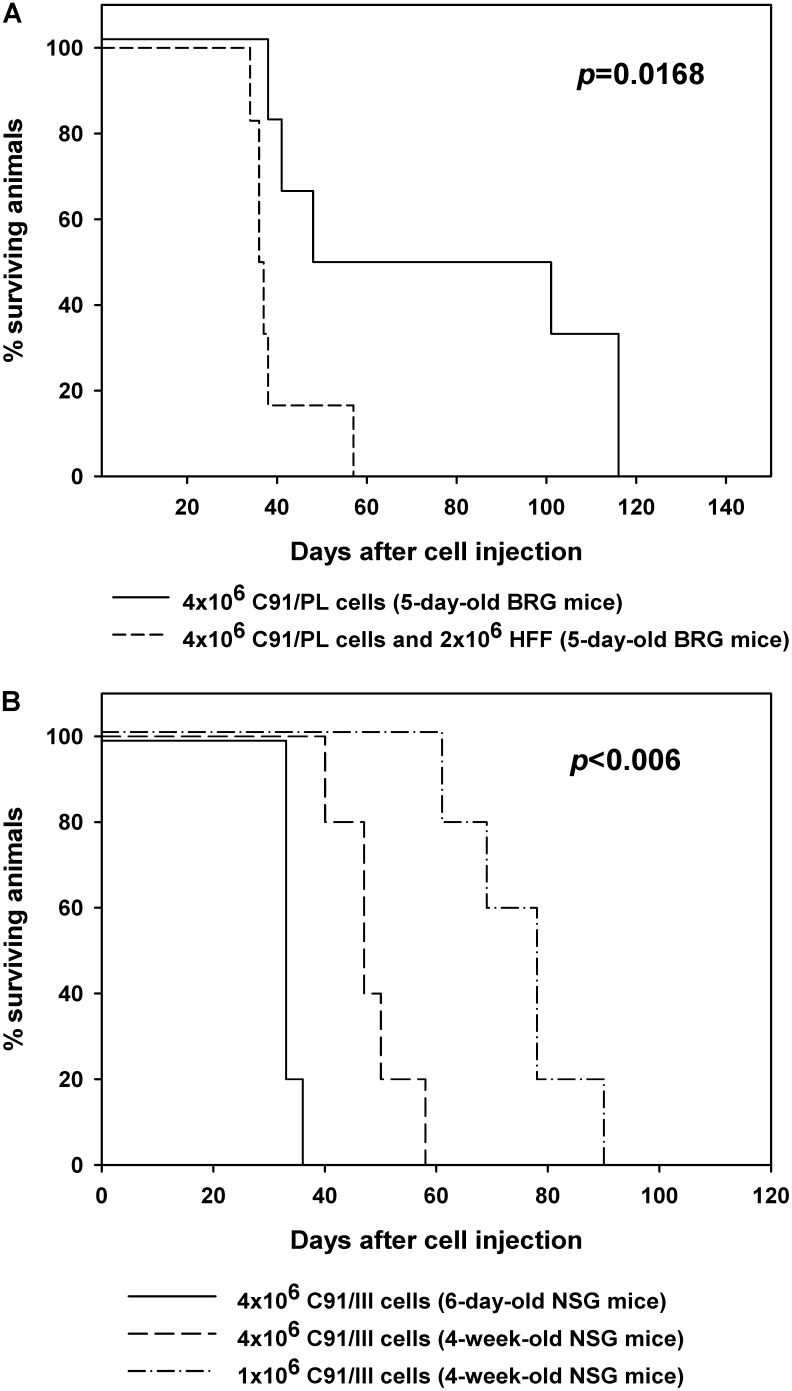
Kaplan–Meier survival curves for mice injected with C91/PL cells, C91/PL cells with human foreskin fibroblasts (HFF), or C91/III cells. **(A)** Five-day-old BRG mice intraperitoneally injected with C91/PL cells and HFF showed a statistically significant decrease in the survival time compared to control mice injected with C91/PL cells (six mice per group). **(B)** Engraftment of C91/III cells in NSG mice. Survival curves for 6-day-old or 4-week-old mice intraperitoneally inoculated with different doses of C91/III cells (five mice per group). *P*-values reported in the figure were calculated by the log-rank test; *p*-value in **B** refers to all pairwise comparisons and was adjusted for multiple comparisons with Bonferroni correction.

To further confirm the supporting activity exerted by fibroblasts, a second set of experiments was performed. As the previously tested Rag2^-/-^γ_c_^-/-^ mice were no longer available in our animal house (see section “Materials and Methods”), we used brand new mice with identical knock-out genes (BALB/c Rag2^-/-^γ_c_^-/-^, BRG), but obtained from a different source and with a well-defined, restricted microbiota, that were endowed with high susceptibility to tumor xenoengraftment (Yamamoto and Schiestl, 2014; Rosshart et al., 2017). The experimental setting was identical to the previous one and two groups of six, 5-day-old, BRG mice were similarly i.p. injected with C91/PL cells alone (control group) or mixed with HFF in a 2:1 ratio. Mice injected with HFF and C91/PL cells developed lymphoma as did control mice injected with C91/PL cells alone (**Figure 2A**). However, HFF co-inoculation significantly triggered lymphoma development compared to control mice, with a twofold decrease in survival time (log-rank test, *p* = 0.0168). The pathological findings of mice developing lymphoma were substantially similar to those observed in the first set of experiments.

Therefore, these data indicate that, in appropriate hosts, C91/PL cells may *per se* induce lymphoma in immunocompromised mice. However, HFF co-inoculation exerts a significant enhancement of the oncogenic potential of C91/PL cells *in vivo*, confirming the relevance of stromal contribution in HTLV-1-mediated lymphomagenesis.

### C91/III Cells Efficiently Engraft in NSG Mice

To further confirm the constitutive capability of C91/III cells to induce lymphoma with high reproducible efficiency, a different mouse strain was employed. Therefore, mice of NSG strain were i.p. injected with C91/III cells. **Figure 2B** shows the Kaplan–Meier survival curves for mice injected with C91/III cells at 6 days or 4 weeks of age (five mice each group). Six-day-old mice injected with 4 × 10^6^ cells had a median survival time (33 days) similar to that of baby Rag2^-/-^γ_c_^-/-^, and showed at necroscopy diffused mesenteric lymphomatous masses; two of them showed macroscopic lung metastatic nodules. Interestingly, the same dose of C91/III cells induced lymphoma in all young adult mice with a median survival time of 47 days. Moreover, even at a dose of 1 × 10^6^ cells, all young adult mice died from a diffused tumor, with a median survival time of 78 days. At necroscopy, diseased mice showed abdominal tumor masses involving the peritoneal and pelvic organs and frequent lung involvement.

These data show that C91/III cells, even in low dose, engraft with high efficiency in NSG mice and induce an aggressive lymphoma with a relative short latency in young adult mice. Therefore, C91/III cells xenotransplanted in the adult NSG mouse setting might usefully be employed for preclinical evaluation of drug candidates for ATLL lymphoma variant.

### Characterization of the Tumorigenic C91/III Cell Line

Based on the results obtained in NSG mice, C91/III cells were chosen as a representative tumorigenic cell line for further characterization. Analysis of clonality showed an identical monoclonal pattern in the rearrangement of the β and γ chains of the TCR gene in C91/PL and C91/III cells (not shown). STR profile of C91/III cells further confirmed derivation from the parental cell line (**Supplementary Table S1**). The presence of the HTLV-1 provirus was confirmed by a qualitative standard PCR using primer pairs specific for the *tax* region (not shown).

**FIGURE 3:**
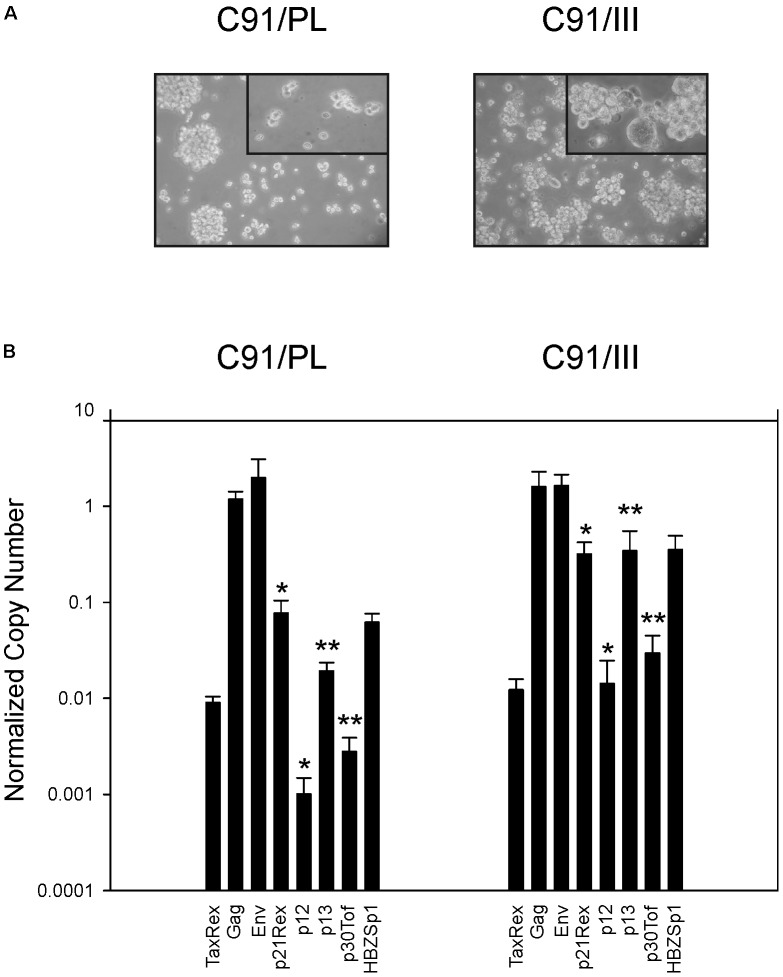
Morphologic and viral characterization of C91/PL and C91/III cell lines. **(A)** Phase contrast images of C91/PL and C91/III cells in standard culture conditions. The tumorigenic C91/III cell line showed a higher percentage of multinucleated giant cells. Original magnification 100×; Upper right-end side insert 200×. **(B)** Profile of Human T cell Leukemia Virus type 1 (HTLV-1) transcripts. C91/III cells showed a statistically significant increase in the normalized copy number (NCN) of all transcripts encoding the viral accessory proteins compared to C91/PL cells. NCN values were calculated by normalizing the absolute copy number of each transcript for the copy number of the glyceraldehyde 3-phosphate dehydrogenase (GAPDH) transcripts. Values are the mean of three independent measurements performed in triplicate; standard error bars are shown. ^∗^*p* < 0.05, ^∗∗^*p* < 0.01.

C91/III cells morphologically resembled C91/PL cells, although the percentage of giant cells was higher (**Figure 3A**). Phenotypic analyses did not disclose substantial differential expression of surface markers (**Table 3**). C91/III cells were all CD4+/CD25+ as the parental C91/PL cell line, they expressed similar levels of CD5 and, at high intensity, the two adhesion molecules CADM1 and CD54. C91/PL and C91/III cells did not stain for stem cell markers (CD34, CD117, and CD133). Intracellular staining for forkhead box P3 (FOXP3) transcriptional factor was similar in the two cell lines.

**Table 3:** Immunophenotypic characterization of C91/PL and C91/III cells.

Marker	C91/PL	C91/III
CD1a	–^a^	–
CD2	–	–
CD3	–	–
CD4	++++^b^	+++/++++
CD5	++/+++	++/+++
CD7	–	–
CD25	+++	++/+++
CD34	–	–
CD117	–	–
CD133	–	–
CD54	++++	++++
CADM1	++++	++++
FOXP3	+++	+++


**^*a*^Negative. ^*b*^+ indicates that <25% of cells expressed the analyzed surface marker by cytofluorimetric analyses; ++ indicates 25–50%; +++ indicates 50–75%; ++++ indicates >75%.**

Comparative qRT-PCR analysis of the viral transcript profile in C91/PL and C91/III cells showed no significant variation in the expression of the gag, env, and tax/rex mRNAs. In contrast, all the transcripts coding for the accessory proteins showed a marked increase in C91/III cells, ranging from 4-fold (p21rex) to 18-fold (p13). Statistical analysis showed that these differences were significant (*p* ≤ 0.05) except for HBZ Sp1 (*p* = 0.056) (**Figure 3B**).

### Characterization of the Secretory Profile of C91/PL, C91/III Cells, and HFF

**FIGURE 4:**
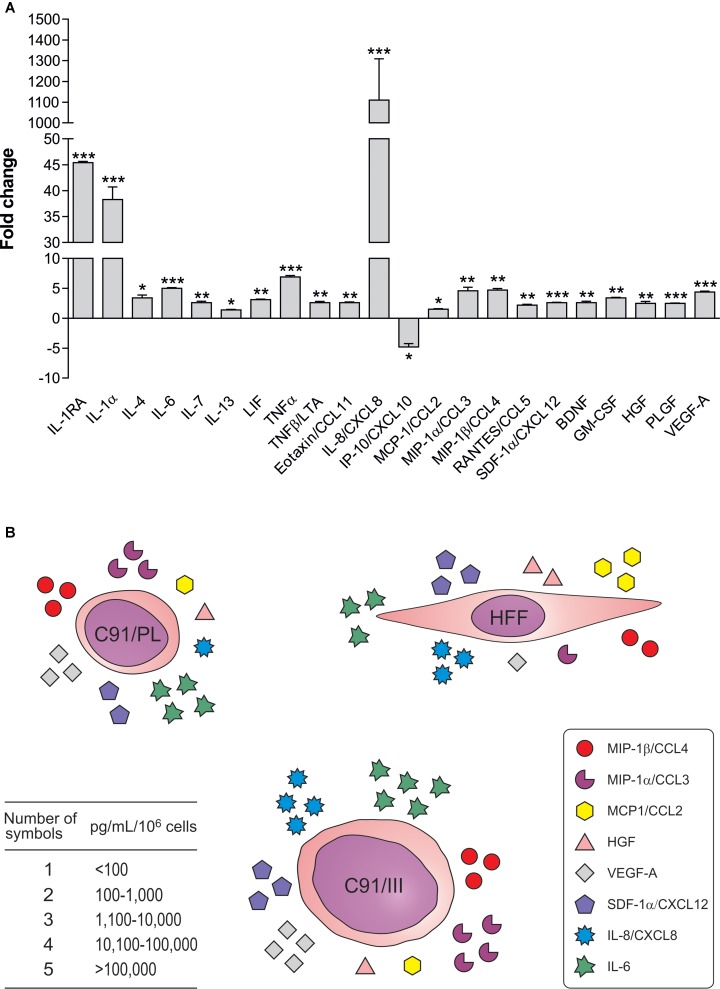
Soluble factors differentially released by C91/III cells. **(A)** Data are reported as ratio between the mean of the values, expressed in pg/mL/10^6^ cells, measured in the supernatants of C91/III cells and in the supernatants of C91/PL cells; standard deviations of the ratio are calculated according to the theory of error propagation (Calabro et al., 2009). Only significantly different soluble factors are shown, and statistical significance is referred to the comparison of the concentration values between the two cell lines, calculated by two-tailed Student’s *t*-test. ^∗^*p* < 0.05, ^∗∗^*p* < 0.01, ^∗∗∗^*p* < 0.001. **(B)** The figure schematically shows that some of the soluble factors increased in the lymphomagenic cells belong to the HFF secretory profile, suggesting that C91/III cells acquired an autocrine stimulatory loop, becoming independent from HFF support.

To assess whether C91/III cells were characterized by a differential profile of secreted soluble factors, we measured 45 cytokines, chemokines and growth factors in the supernatants of both cell lines (**Supplementary Table S2**). The secretory profile of C91/III cells did not qualitatively differ from that of the parental cell line, at least in the 45 soluble factors tested using the Luminex technology. However, the release of 22 factors was quantitatively altered in C91/III cells, as shown in **Figure 4A**. The amount of 21 soluble factors was significantly increased in C91/III cells, while only one (IP-10/CXCL10) was reduced. Many of the upregulated factors were direct pro-inflammatory cytokines (IL-1α, IL-6, TNFα) and α and β chemokines (**Supplementary Table S2**), able to amplify the inflammatory response. Interestingly, the highest increase was shown by IL-8/CXCL8, a chemokine involved in tumorigenesis through autocrine and paracrine signaling responsible for the triggering of invasiveness, angiogenesis and metastasis (Balkwill and Mantovani, 2001).

We also characterized the secretory pattern of HFF used in our *in vivo* experiments (**Supplementary Table S2**). The high release of many chemokines and IL-6 indicated that HFF partially overlapped the secretory profile of “activated” fibroblasts (Kalluri and Zeisberg, 2006). Among the growth factors, HGF, βNGF, and VEGF-A were found in measurable amounts, while EGF, FGF-2, and PDGF-BB, which are other soluble mediators secreted by activated fibroblasts, were not detected. Interestingly, some of the factors released by C91/III cells showing the most pronounced increase were characteristic of the HFF secretory profile (**Figure 4B**) suggesting that C91/III cells might have reinforced autonomous stimulatory activity, thus becoming independent from the microenvironment support.

### Analysis of the Crosstalk Between C91/PL Cells and HFF

**FIGURE 5:**
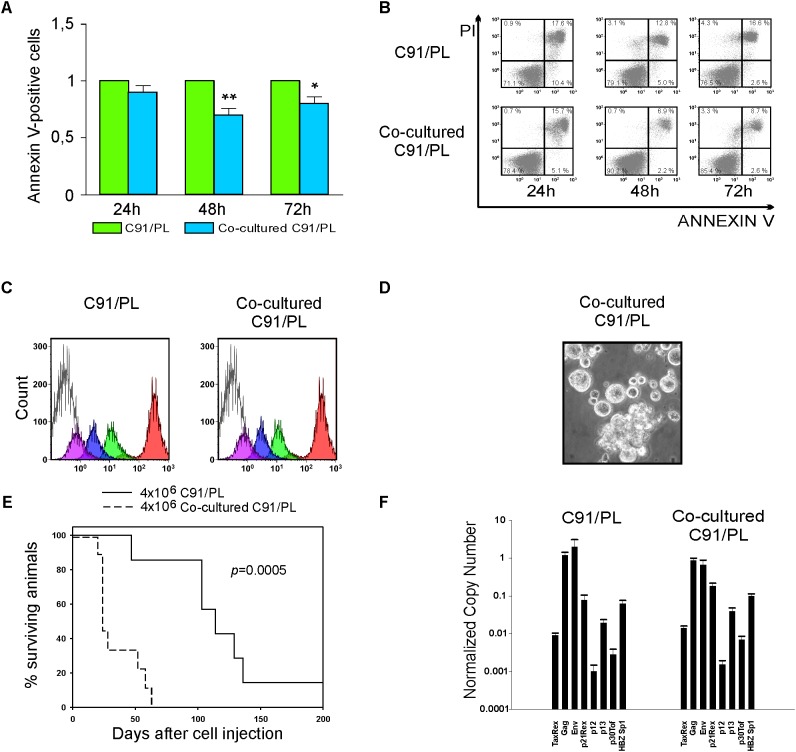
Analysis of the *in vitro* crosstalk of C91/PL cells and HFF. **(A)** Co-culture with HFF reduced apoptosis in C91/PL cells. Apoptosis was analyzed in C91/PL cultured in standard conditions and in C91/PL cells co-cultured with HFF cells at 24, 48, and 72 h. Data are reported as ratio between the mean of the percentage of Annexin V–positive cells in two different co-cultures and the standard deviations (SD) of the ratio are calculated among four different experimental groups. Data are presented as mean ± SD. SD of the ratio was calculated according to the theory of error propagation. Statistical significance was determined by two-tailed Student’s *t*-test. ^∗^*p* < 0.05, ^∗∗^*p* < 0.01. **(B)** Flow cytometry analysis of Annexin V/Propidium Iodide (PI) stained C91/PL cells cultured in standard conditions **(Upper)** and in co-culture with HFF **(Lower)** at three different time points. A representative experiment is shown and the percentage of cells in each quadrant of the flow plots is provided. **(C)** Measurement of the *in vitro* proliferation of C91/PL cells cultured in the absence **(Left)** or presence **(Right)** of HFF. Cells were labeled with carboxyfluorescein succinimidyl ester (CFSE) and analyzed with XL Epic cytofluorimeter after 0 h (red), 24 h (green), 48 h (blue), and 72 h (violet). The shaded histogram shows the unlabeled cells. The plots show that co-culture with HFF does not affect the proliferation rate of C91/PL cells. Histograms represent the data obtained in two independent experiments. **(D)** Phase contrast images of C91/PL cells after co-culture with HFF. Eight-week-co-culture with HFF induced an increase in giant cells, thus resembling the C91/III cell line. Original magnification 100×. **(E)** Kaplan–Meier survival curves for 5-day-old NSG mice i.p. injected with 4 × 10^6^ C91/PL cells (seven mice) and with 4 × 10^6^ co-cultured C91/PL cells (nine mice). A statistically significant reduction (log-rank test; *p* = 0.0005) in the overall survival of mice injected with co-cultured cells was observed. **(F)** Analysis of HTLV-1 transcripts. Quantitative analyses of viral transcripts showed no significant variation in any of the HTLV-1 mRNAs in co-cultured C91/PL cells compared to the C91/PL cell line, suggesting that the increased survival and *in vivo* lymphoma induction acquired by co-cultured cells were not likely due to virus-encoded factors. Data are reported as described in **Figure 3B**.

As HFF were found to trigger tumorigenesis of C91/PL cells, we studied the impact of HFF on C91/PL cells in a co-culture system. Results showed that co-cultivation with HFF significantly decreased the induced apoptosis in C91/PL cells after 48 and 72 h of co-culture (**Figures 5A,B**). On the other hand, HFF-co-cultured C91/PL cells showed proliferation rates comparable to those of C91/PL cells, as assessed by CFSE labeling (**Figure 5C**). Of interest, short-term and long-term co-culture with HFF led to morphologic changes in C91/PL cells resembling those of C91/III cells (**Figure 5D**, see **Figure 3A** for comparison), whereas immunophenotypic variations were not observed for the periodically checked surface antigens (CD4, CD25, and CD54, data not shown).

*In vivo* lymphomagenic activity was analyzed in 5-day-old NSG mice injected i.p. with co-cultured C91/PL cells, which showed a statistically significant reduction (log-rank test, *p* = 0.0005) in the survival compared to control mice i.p. injected with C91/PL cells (**Figure 5E**).

On the other hand, no significant variation in the expression of any of the viral mRNAs was detected (**Figure 5F**), suggesting that the co-culture microenvironment does not have an impact on either HTLV-1 rate of transcription or on the alternative splicing pattern of viral mRNAs.

Interestingly, the secretory pattern of co-cultured C91/PL cells was similar, albeit not identical, to that found in C91/III cells (**Supplementary Figure S2**), with a statistically significant increase in IL-8/CXCL8 and many other chemokines. Among pro-inflammatory factors, a significant increase was evidenced for TNFα, whereas IL-1α and IL-6 were augmented but not at a statistically significant level.

To assess the relevance of the paracrine crosstalk between C91/PL cells and HFF, two soluble factors were measured after 3 and 10 days of co-culture in direct contact or in transwell inserts. Transwell co-culture induced an increase, even though less pronounced, in secreted IL-8/CXCL8 and TNFα (**Supplementary Figure S3**), suggesting that soluble crosstalk is also implicated in this heterotypic cell interaction.

On the whole, these data indicated that HFF may increase the survival of C91/PL cells *in vitro* and may contribute to the induction of morphological and secretory changes similar to those of C91/III cells. Most importantly, co-cultured C91/PL cells were much more tumorigenic compared to the parental cells when injected into baby NSG mice. On the other hand, the remarkably overlapping viral expression patterns of C91/PL cells, with or without HFF, argue against a role of viral genes in the *in vivo* more aggressive behavior of co-cultured C91/PL cells and, likely, of C91/III cells.

## Discussion

To recapitulate ATLL pathogenesis *in vivo*, transgenic and humanized mouse models have been developed. Tax-transgenic mice mainly developed arthropathy and other inflammatory disorders (Iwakura et al., 1991; Yamamoto et al., 1993; Saggioro et al., 1997) or solid tumor (Hinrichs et al., 1987; Nerenberg et al., 1987) and leukemia (Grossman et al., 1995; Hasegawa et al., 2006; Ohsugi, 2013), depending on the promoter used. HBZ-transgenic mice develop not only T-cell lymphoma but also systemic chronic inflammation after a long latency period and with low/variable incidence (Satou et al., 2011). Thus, transgenic mice are useful tools to investigate the activities of the Tax and HBZ proteins but are limited in that the resulting phenotypes are dependent on a given promoter and the functions of other viral products are missing.

Further ATLL models exploiting the humanized mice were recently developed, based on immunodeficient animals transplanted with human hematopoietic stem cells from cord blood or fetal liver, thus able to reconstitute a human immune system permissive to HTLV-1 infection. Villaudy et al. (2011) generated humanized mice by intrahepatic engraftment of human cord blood CD34^+^ cells into Rag2^-/-^γ_c_^-/-^ mice. During thymocyte maturation, these mice were infected with intraperitoneal injection of irradiated MT-2 cells as virus donors. Some of the animals, after a long latency, showed pathological signs resembling the lymphoma variant of ATLL. A different clinical presentation was achieved in HTLV-1-infected humanized NOG mice (Tezuka et al., 2014), showing ATLL-like leukemogenesis, characterized by leukocytosis, hepatosplenomegaly, high plasmatic levels of cytokines and sporadic appearance of flower cells in peripheral blood. The humanized mouse model, although promising for investigating the natural history of HTLV-1 infection and the host-specific immune response, critical steps for eventual development of ATLL, is however not easily achievable because of the rather complex procedures including the ethics committee evaluation for the use of human primary hematopoietic stem cells and fetal tissues.

Engraftment of *in vitro* HTLV-1-infected and immortalized T cells gave variable results in SCID mice, depending on which cell line was tested (Feuer et al., 1993; Imada et al., 1995), and was shown to be facilitated by abrogation of the host NK function obtained through irradiation or treatment with an anti-asialo GM1 antibody (Ishihara et al., 1992; Feuer et al., 1995).

Subsequent experiments on xenotransplantation of *in vitro* HTLV-1-transformed T cell lines or peripheral blood mononuclear cells from HTLV-1-infected asymptomatic carriers in mice with various degrees of immunodeficiency confirmed that lack of innate immunity is crucial for a favorable neoplastic infiltration and/or outgrowth (Liu et al., 2002; Takajo et al., 2007). This finding is consistent with the beneficial effect exerted by alloreactive donor NK cells, generated following hematopoietic stem cell transplantation, on the clinical outcome of leukemia patients (Gismondi et al., 2015).

Therefore, to focus on the influence of host microenvironment other than immune cell components, BALB/c Rag2^-/-^γ_c_^-/-^ and BRG (“excluded flora”) mice, characterized by immunological dysfunction of T, B, and NK cells, were employed in the present study. By injecting intraperitoneally newborn mice with C91/PL cells together with HFF a significant triggering of lymphoma development was observed in two sets of experiments (**Table 1** and **Figure 2A**). The different percentage of mice developing lymphoma in the two series of experiments may likely be explained by mouse intra-strain genetic background variation (Eisener-Dorman et al., 2009) and/or different gut microbiota (Yamamoto and Schiestl, 2014; Rosshart et al., 2017). Unfortunately, experiments could not be continued in the Rag2^-/-^γ_c_^-/-^ mice first employed in our experiments, as these animals are no longer available. C91/PL-derived cell lines were further established, which consistently induced neoplastic infiltration when injected alone in newborn and young adult Rag2^-/-^γ_c_^-/-^ and NSG mice (**Table 2** and **Figure 2B**). This indicated that a stable and highly tumorigenic capacity was acquired by C91/PL cells following co-transplantation with HFF and subsequent *in vitro/in vivo* passages. Immunodeficient mice xenotransplanted with C91/I, C91/II, and C91/III cell lines exhibited lymphomatous masses diffused to lungs, abdominal and pelvic organs, reminiscent of the lymphoma variant of ATLL (**Figure 1**). Their engraftment efficiency was more reproducible and shorter in latency than that exhibited by the parental C91/PL cells. Interestingly, the frequent lymphomatous involvement of lungs in mice injected with C91/II and C91/III cells indicates an increased lung tropism, a peculiar feature of ATLL cells. In fact, pulmonary chronic inflammatory lesions were described in about 30% of HTLV-1 carriers and lung lymphomatous infiltration was observed in 45% of ATLL patients (Yoshioka et al., 1985; Okada et al., 2006).

The role of stromal microenvironment, and particularly of fibroblasts, its major cellular component, on tumor induction has been extensively documented in epithelial cancers, leading to the notion that fibroblasts exhibit a bimodal effect (Bhowmick et al., 2004; Kalluri and Zeisberg, 2006). In the initial phase of tumorigenesis, they constrain the oncogenic conversion by remodeling the stromal architecture, while in later stages they promote tumor progression and invasiveness, engaging an active crosstalk with the cancerous cells (Marsh et al., 2013; Heneberg, 2016). Complex crosstalk between bone marrow microenvironment and leukemia cells has also been reported, and therapeutic interventions targeting leukemia niches have been proposed (Jin et al., 2008; Zhang et al., 2012). Similarly, lymphoma cells may interact with stromal components to acquire a more aggressive and invasive behavior. In the setting of ATLL, co-culture of clinical samples of ATLL cells, *in vitro* HTLV-1-transformed and ATLL-derived cell lines with murine bone marrow stromal cells supported the growth of primary ATLL cells as well as established cell lines (Nagai et al., 2008). The proliferative enhancement was paralleled by downregulation of Tax, while the expression level of HBZ gene remained unchanged (Nagai et al., 2008). Kinpara et al. (2009) also found that co-culture of ATLL-derived or HTLV-1-transformed T cells with human epithelial-like cells (HEK293T cells) or with murine NIH3T3 fibroblasts did suppress viral p19 and *gag* mRNA, and this effect, albeit reversible, was mediated by IFN-α and IFN-β. ATLL cell lines and primary ATLL cells, after short-term co-culture with epithelial-like cells were found to be protected from apoptosis, became quiescent and acquired a cancer stem cell-like phenotype (Miyatake et al., 2013, 2015). All these findings highlight the relevant impact of microenvironment in ATLL pathogenesis, indicating that stromal signaling may contribute to the establishment of viral latency as well as may induce *in vitro* a more aggressive neoplastic phenotype. In the present study we show the role of human primary fibroblasts in T-cell lymphomagenesis using an *in vivo* preclinical setting.

Preliminary characterization of the lymphomagenic C91/III cell line, derived from the parental C91/PL cells, did not show relevant phenotypic changes (**Table 3**), but the analysis of its secretory repertoire indicated that mechanisms involved in epithelial cancers may also apply to ATLL. Indeed, a relevant role in lymphomagenesis is played by cytokines and chemokines which provide a milieu favorable to tumor growth and a signaling path for cell migration and tissue invasion (Hsu et al., 1993). The disseminated nature of the neoplasia developed in C91/III-injected mice was paralleled by a substantial alteration in the secretory profile of these cells, mainly characterized by the significant increase of several soluble factors, most of which equipped with a direct (IL-1α, IL-6, TNFα) or indirect (mainly chemokines) pro-inflammatory activity (**Figure 4**). The major fold increment was found in IL-8/CXCL8, an important mediator for tissue infiltration (Balkwill and Mantovani, 2001). Remarkably, some of the factors increased in C91/III cells also belonged to the secretory profile of ATLL cells (Yamada et al., 1996) as well as of HFF, and are known to facilitate tumor progression *in vivo* (Davalos et al., 2010). These data suggest that C91/III cells may autonomously induce a pro-inflammatory-like status that, through autocrine and paracrine loops, is able to promote tumorigenesis and organ infiltration. Interestingly, the *in vitro* crosstalk of C91/PL cells with HFF led to the acquisition of a pro-inflammatory secretory phenotype similar to that observed in the tumorigenic C91/III cells (**Supplementary Figure S2**). HFF-co-cultured C91/PL cells not only changed their turn over, acquiring resistance to induced apoptosis (**Figures 5A,B**) but, most importantly, acquired an enhanced lymphomagenic capability upon xenotransplantation (**Figure 5E**).

Transwell experiments also demonstrated a moderate increase in IL-8/CXCL8 and TNFα secretion, thus suggesting that, in addition to cell-to-cell contact, soluble factors play a role in the heterotypic cell interaction (**Supplementary Figure S3**). However, it should be mentioned that *in vivo* preliminary experiments by co-injection of conditioned medium from HFF with C91/PL cells failed to increase cell tumorigenesis (data not shown). Additional studies are needed to further confirm that HFF-secreted factors are not sufficient *per se* to modify the *in vivo* behavior of C91/PL cells. The overall increase in the expression of all viral accessory mRNAs observed in C91/III cells (**Figure 3B**) might suggest the acquisition of defective proviruses during the process of clonal evolution of these cells, and denotes another feature reminiscent of ATLL cells *in vivo* (Ohshima et al., 1991). However, HFF-co-cultured C91/PL cells showed no significant differences in the viral transcriptional profile (**Figure 5F**), suggesting that the increased tumorigenicity of co-cultured cells, and likely of C91/III cells, is not driven by viral factors.

Evolutionary models of cancer development imply the existence of silent precursor cells from which, through a stepwise process of accumulation of somatic mutations, aggressive malignancies eventually rise (Greaves, 2015). Actually, persistence of the pre-leukemic precursors, carrying a founder mutation, albeit not malignant *per se*, has recently been demonstrated in acute leukemia (Shlush et al., 2014). In the ATLL setting, it may be envisaged that the insertion of the HTLV-1 DNA provirus in the genome of T cells constitutes the “founder” mutation conferring them the properties of persistent, pre-malignant ancestors, which, upon additional favorable forces, may ultimately reach full oncogenic capacity. A well-known similar condition is represented by the long life persistence of B cells immortalized by the Epstein-Barr virus from which lymphomas may develop in immunosuppressed subjects.

## Conclusion

The finding that fibroblasts possessing an activated-like, pro-inflammatory secretory phenotype enhance the tumorigenesis of C91/PL cells throws light on the triggering activity of the host microenvironment on HTLV-1-linked lymphomagenesis. More work is needed to clarify the basis of this triggering effect as well as to reveal whether genetic and epigenetic changes, produced within the HTLV-1-immortalized T cells, share the responsibility for full-blown malignancy.

Furthermore, our results show that the C91/III cell line, originating from a long-term *in vitro* HTLV-1-immortalized T cell line, is endowed with a highly reproducible lymphomagenic capacity in NSG mice that may be exploited as a simple murine preclinical model to investigate *in vivo* the efficacy of therapeutic interventions against the lymphoma variant of ATLL.

## Author Contributions

MV and AM designed the experiments, performed the research and data analysis, and contributed to paper writing. BM and MAP performed the research, analyzed the results, and contributed to paper writing. AA contributed to the interpretation of data and critically reviewed the paper. IC performed the research and data analysis for HTLV-1 transcriptional profiles, and critically revised the paper. LC-B conceived and designed the study, established the preclinical model, analyzed the results, and wrote the paper. MLC conceived and designed the study, established the new cell lines, analyzed the results, and wrote the paper.

## Conflict of Interest Statement

The authors declare that the research was conducted in the absence of any commercial or financial relationships that could be construed as a potential conflict of interest.

## Supplementary Material

FIGURE S1 Representative photomicrographs of immunoreactions for CD25 showing lymphomatous infiltration in the lung parenchyma **(A)** and a nearby pulmonary bronchiole **(B)** observed in a C91/III cell-injected mouse. Original magnification 400×.

FIGURE S2 Soluble factors released by C91/PL cells after co-culture with HFF. Cytokines, chemokines and growth factors secreted in culture supernatants by C91/PL cells removed after 2 months from the co-culture with HFF and maintained as long as 6 weeks in culture. Values are normalized to those of C91/PL cells. Data are reported as ratio between the mean of the values, expressed in pg/mL/10^6^ cells, measured in co-cultured C91/PL cells and the mean of the values measured in the supernatants of C91/PL cells, and the standard deviations of the ratio are calculated according to the theory of error propagation (Calabro et al., 2009). Only significantly different soluble factors are shown, and statistical significance was determined by two-tailed Student’s *t*-test. ^∗^*p* < 0.05, ^∗∗^*p* < 0.01, ^∗∗∗^*p* < 0.001.

FIGURE S3 IL-8/CXCL8 and TNFα released in culture supernatants after short-term co-culture of C91/PL cells with HFF. A significant increase in the secretion of IL-8/CXCL8 **(A)** and TNFα **(B)** was observed after 3 days of co-culture of C91/PL cells, either in direct contact or placed in transwell inserts, with HFF; IL-8/CXCL8 increment persisted after 10 days of co-culture. Control wells with C91/PL cells or HFF were set up and analyzed in parallel. As previously observed (**Supplementary Table S2**), HFF did not contribute to TNFα increase. The increase in IL-8/CXCL8 and TNFα in transwell co-cultures, albeit lower than that measured in direct co-cultures, indicated that the heterotypic crosstalk is also mediated by soluble factors. Data are expressed in pg/mL/10^6^ cells. Statistical significance was calculated by two-tailed Student’s *t*-test. ^∗^*p* < 0.05; ^∗∗^*p* < 0.01; ^∗∗∗^*p* < 0.001; ^∗∗∗∗^*p* < 0.0001.

TABLE S1 Short Tandem Repeat (STR) profiles of cell lines.

TABLE S2 Soluble factors released by C91/PL and C91/III cells and human foreskin fibroblasts (HFF).
