# Supplementary figures and images for: A Preclinical Model for the ATLL Lymphoma Subtype With Insights Into the Role of Microenvironment in HTLV-1-Mediated Lymphomagenesis
Source: Front Microbiol. 2018 Jun 13;9:1215. doi: 10.3389/fmicb.2018.01215 (PMC6008390; doi:10.3389/fmicb.2018.01215)

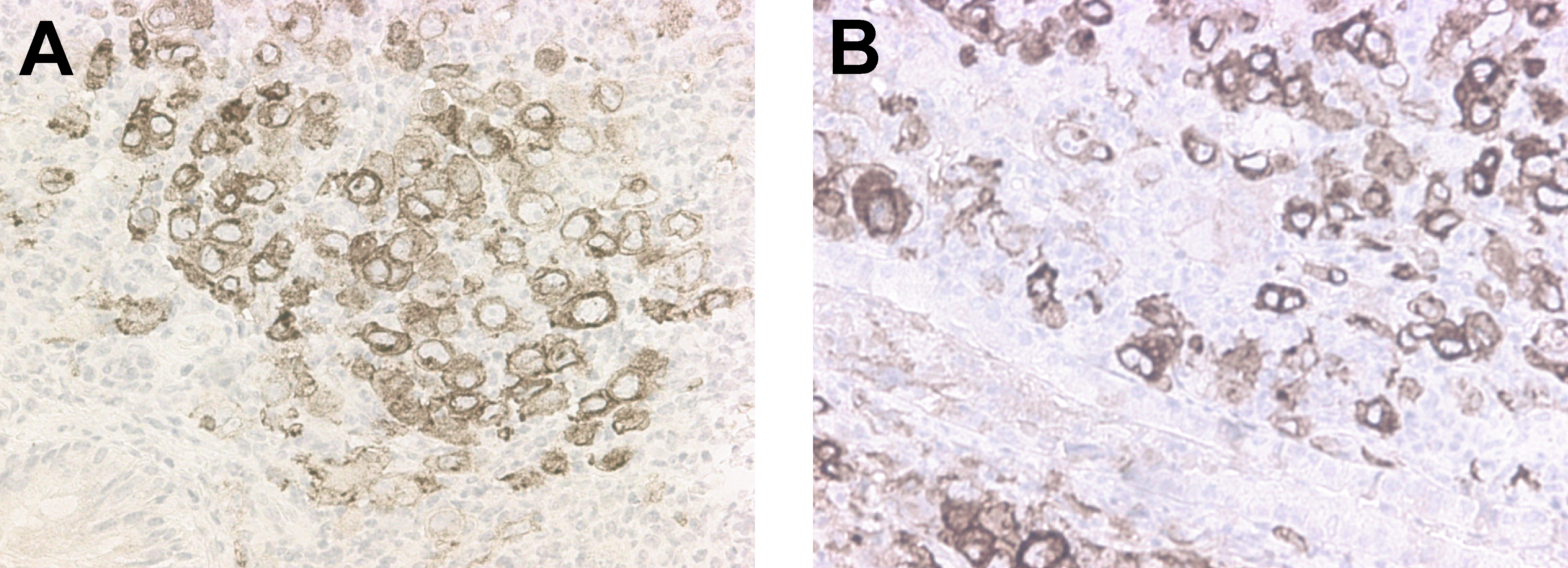

Supplement: FIGURE S1 — Representative photomicrographs of immunoreactions for CD25 showing lymphomatous infiltration in the lung parenchyma (A) and a nearby pulmonary bronchiole (B) observed in a C91/III cell-injected mouse. Original magnification 400×. [file Image_1.TIF]

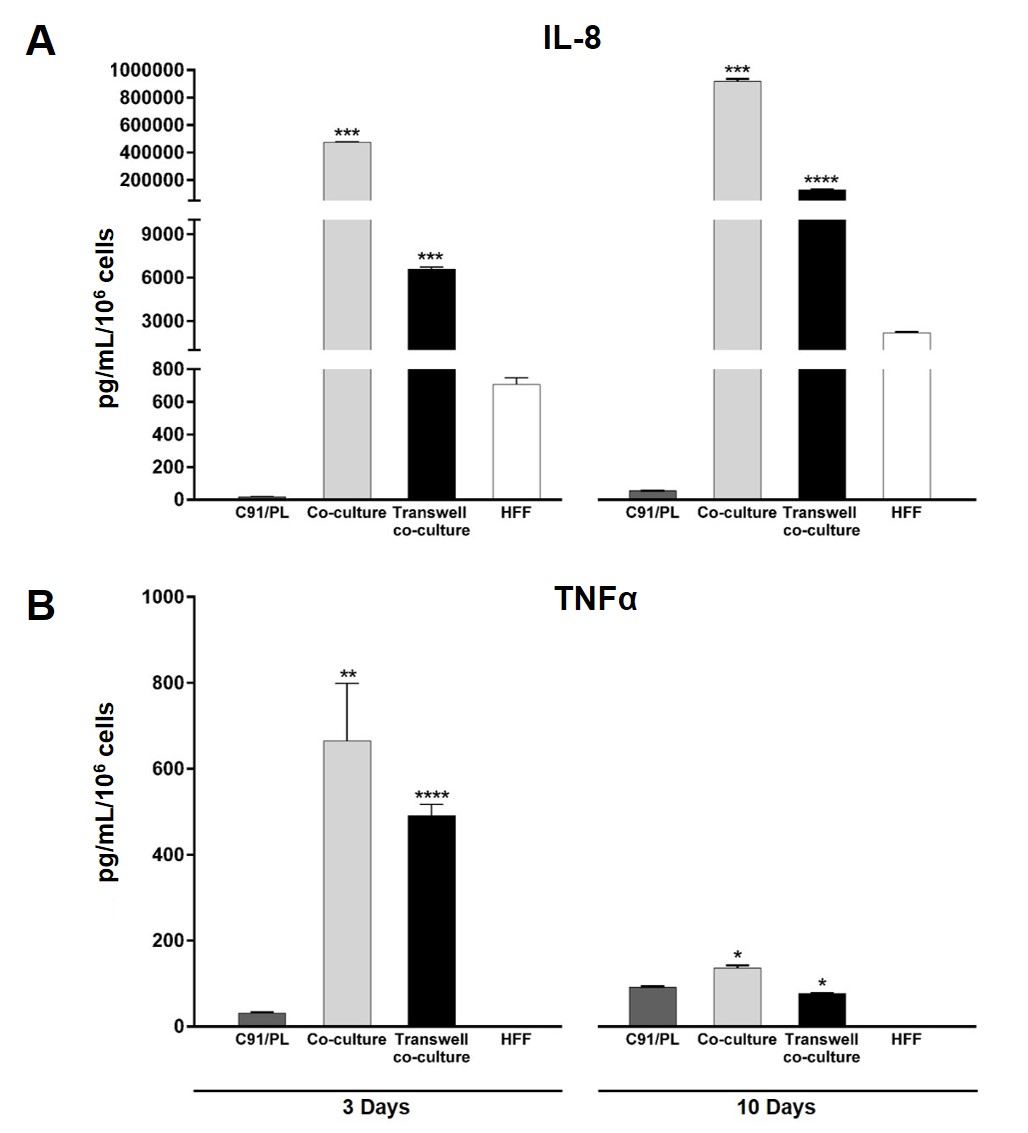

Supplement: FIGURE S3 — IL-8/CXCL8 and TNFα released in culture supernatants after short-term co-culture of C91/PL cells with HFF. A significant increase in the secretion of IL-8/CXCL8 (A) and TNFα (B) was observed after 3 days of co-culture of C91/PL cells, either in direct contact or placed in transwell inserts, with HFF; IL-8/CXCL8 increment persisted after 10 days of co-culture. Control wells with C91/PL cells or HFF were set up and analyzed in parallel. As previously observed (Supplementary Table S2), HFF did not contribute to TNFα increase. The increase in IL-8/CXCL8 and TNFα in transwell co-cultures, albeit lower than that measured in direct co-cultures, indicated that the heterotypic crosstalk is also mediated by soluble factors. Data are expressed in pg/mL/106 cells. Statistical significance was calculated by two-tailed Student’s t-test. ∗p < 0.05; ∗∗p < 0.01; ∗∗∗p < 0.001; ∗∗∗∗p < 0.0001. [file Image_3.JPEG]
