# Supplementary material for: A Preclinical Model for the ATLL Lymphoma Subtype With Insights Into the Role of Microenvironment in HTLV-1-Mediated Lymphomagenesis
Source: Front Microbiol. 2018 Jun 13;9:1215. doi: 10.3389/fmicb.2018.01215 (PMC6008390; doi:10.3389/fmicb.2018.01215)
Supplement: TABLE S1 — Short Tandem Repeat (STR) profiles of cell lines. [file Table_1.PDF]

SUPPLEMENTARY TABLE 1 | Short Tandem Repeat (STR) profiles of cell lines

Marker	C91/PL	C91/II	C91/III	C91/PL (UK)
D3S1358	15-19	15-19	15-19	15-19
TH01	8-9	8-9	8-9	8-9
D21S11	29-31.2	29-31.2	29-31.2	29-31.2
D18S51	12-13	12-13	12-13	12-13
Penta E	16-20	16-20	16-20	16-20
D5S818	13	13	13	13
D13S317	8-11	8-11	8-11	11
D7S820	10-11	10-11	10-11	10-11
D16S539	9-12	9-12	9-12	9-12
CSF1PO	10	10	10	10
Penta D	10-13	10-13	10-13	10-13
AMEL	X-Y	X-Y	X-Y	X-Y
vWA	17	17	17	17
D8S1179	13-14	13-14	13-14	13-14
TPOX	8	8	8	8
FGA	19-23	19-23	19-23	19-23
D19S433	13-15	13-15	13-15	13-15
D2S1338	21-25	21-25	21-25	21-25
