# Supplementary material for: A Preclinical Model for the ATLL Lymphoma Subtype With Insights Into the Role of Microenvironment in HTLV-1-Mediated Lymphomagenesis
Source: Front Microbiol. 2018 Jun 13;9:1215. doi: 10.3389/fmicb.2018.01215 (PMC6008390; doi:10.3389/fmicb.2018.01215)
Supplement: TABLE S2 — Soluble factors released by C91/PL and C91/III cells and human foreskin fibroblasts (HFF). [file Table_2.PDF]

SUPPLEMENTARY TABLE 2 | Soluble factors released by C91/PL and C91/III cells and HFF.

Soluble Factor	C91/PL	C91/III	HFF
IL-1RA	24.92 ± 0.11	1130.74 ± 8.27	< OOR[a]
IL-1α	23.12 ± 1.98	884.40 ± 23.37	< OOR
IL-1β	< OOR	< OOR	< OOR
IL-2	< OOR	< OOR	< OOR
IL-4	65.73 ± 10.82	222.14 ± 26.23	< OOR
IL-5	< OOR	< OOR	< OOR
IL-6	36144.50 ± 1214.10	180125.50 ± 2563.26	10087.86 ±228.71
IL-7	5.09 ± 0.66	13.35 ± 0.55	15.39 ± 0.43
IL-8/CXCL8	83.46 ± 21.13	92631.00 ± 1755.04	7903.18 ± 631.62
IL-9	< OOR	< OOR	< OOR
IL-10	< OOR	< OOR	< OOR
IL-12p70	< OOR	< OOR	< OOR
IL-13	4921.25 ± 384.91	6959.22 ± 49.99	< OOR
IL-15	< OOR	< OOR	< OOR
IL-17A	< OOR	< OOR	< OOR
IL-18	< OOR	< OOR	< OOR
IL-21	< OOR	< OOR	< OOR
IL-22	< OOR	< OOR	< OOR
IL-23	< OOR	< OOR	< OOR
IL-27	< OOR	< OOR	< OOR
IL-31	< OOR	< OOR	< OOR
LIF	283.93 ± 0.39	866.11 ± 37.96	272.97 ± 0.24
TNFα	118.89 ± 5.69	820.40 ± 14.74	< OOR
TNFβ/LTA	898.65 ± 95.66	2313.35 ± 38.78	< OOR
IFNα	< OOR	< OOR	< OOR
IFNγ	< OOR	< OOR	< OOR
GROα/CXCL1	< OOR	< OOR	1029.79 ± 42.52
Eotaxin/CCL11	7.19 ± 0.54	18.41 ± 0.11	223.46 ± 4.38
IP-10/CXCL10	2350.10 ± 389.86	492.12 ± 0.23	10.76 ± 0.61
MCP-1/CCL2	16.22 ± 0.91	24.08 ± 1.52	4310.07 ± 50.81
MIP-1α/CCL3	3702.14 ± 685.78	16905.85 ± 0.12	30.71 ± 1.36
MIP-1β/CCL4	1227.83 ± 97.55	5824.96 ±23.99	988.60 ± 5.44
RANTES/CCL5	174.35 ± 17.61	379.66 ± 0.93	9.92 ± 0.47
SDF-1α/CXCL12	810.17 ± 25.17	2126.21 ± 23.96	3277.69 ± 145.35
BDNF	9.79 ± 1.22	25.70 ± 0.01	365.06 ± 38.34
GM-CSF	6249.46 ± 64.36	21164.67 ± 730.57	< OOR
HGF	27.81 ± 5.06	68.95 ± 0.72	229.06 ± 4.95
PLGF	35.16 ± 0.91	86.43 ± 1.97	< OOR
EGF	< OOR	< OOR	< OOR
FGF-2	< OOR	< OOR	< OOR
PDGF-BB	< OOR	< OOR	< OOR
SCF	< OOR	< OOR	40.67 ± 6.80
βNGF	< OOR	< OOR	168.96 ± 2.14
VEGF-A	4635.44 ± 158.71	20269.61 ± 468.13	48.26 ± 3.52
VEGF-D	< OOR	< OOR	< OOR

[a] Out of range. Data are expressed in picograms/mL/10^6 cells and are reported as the mean ± standard deviations of technical replicates.
